# Pathological Manifestations Rendering Pigs and Cattle Unfit for Transportation in Denmark

**DOI:** 10.3390/ani16030394

**Published:** 2026-01-27

**Authors:** Amanda Øpstun Birk, Henrik Elvang Jensen

**Affiliations:** Faculty of Health and Medical Sciences, Department of Veterinary and Animal Sciences, University of Copenhagen, Ridebanevej 3, 1870 Frederiksberg, Denmark; amanda.birk@sund.ku.dk

**Keywords:** animal welfare, forensic, transport fitness, pigs, cattle

## Abstract

EU regulations concerning the evaluation of animals’ fitness for transportation are in several ways rather vague, which leaves room for individual interpretation of the legislation with a potential negative impact on animal welfare. In order to guide interpretation of the unclear statements within the legislation, all forensic case files handled during a 10-year period (2014–2023) on Danish pigs and cattle that had been declared unfit for transportation and subsequently reported to the police, were evaluated. A total of 327 case files addressing 428 animals were examined. Most pigs were declared unfit for transportation due to the presence of severe open wounds, whereas lameness was the main reason for cattle being deemed unfit. In conclusion, most wounds rendering both pigs and cattle unfit for transportation measured at least 3 cm in diameter, apart from those located in sensitive areas, e.g., tails. Manifestations rendering pigs unfit for transportation due to lameness were primarily joint lesions, whereas fractures were the dominant cause of lameness in cattle. Therefore, the focus should be on these manifestations when assessing animals for transport fitness.

## Simple Summary

Legislation concerning the transportation of animals is overly vague with a potential negative impact on animal welfare. Most wounds rendering pigs and cattle unfit for transportation measured at least 3 cm in diameter unless located in sensitive areas. Pathological manifestations rendering pigs unfit for transportation due to lameness were primarily joint lesions. Pathological manifestations rendering cattle unfit for transportation due to lameness were primarily fractures.

## 1. Introduction

The transport of animals in the EU is regulated by Council Regulation (EC) No 1/2005, which states that “no animal shall be transported unless it is fit for the intended journey, and all animals shall be transported in conditions guaranteed not to cause them injury or unnecessary suffering” [1]. However, in the text it is also stated that sick or injured animals may be considered fit for transport, if they are “slightly injured or ill and transport would not cause additional suffering” [1]. In the EU regulation, a list of three relevant pathological conditions and physiological processes is given that, in particular, makes an animal unfit for transport: (1) animals with a severe open wound, (2) animals with a prolapse, and (3) animals unable to move independently without pain or to walk unassisted [1]. The presence of a prolapse is well defined [1]; however, it is not specified what constitutes a “severe open wound” and the formulations in the regulation are rather vague as “slightly injured or ill” is also not specified. These unclear definitions have led to individual interpretation and potentially negative repercussions for animal welfare [2]. Therefore, in Denmark, guidelines have been elaborated for the assessment of transport fitness by both private organizations [3,4,5,6,7] and public authorities [8]. In the guidelines, examples with respect to different categories, e.g., lameness, hernias, tail bites, and ear wounds in pigs are given together with illustrations for when an animal is (1) fit for transport, (2) fit for transport when certain precautions are taken (e.g., soft bedding and extra space), or (3) not fit for transport. In Denmark, it has been further specified that pigs with large hernias (>15 cm in diameter) causing complications such as restricted mobility or affected general condition, as well as hernias of any size with a wound, are unfit for transportation [9]. Moreover, pigs with large hernias without complications may be transported when separated in groups of up to five animals with the same pathological condition on soft bedding. Furthermore, they must be accompanied by a certificate from a veterinarian deeming them fit for transportation [9].

Except for the well-defined conditions making animals unfit for transport, in many situations it may be difficult to determine whether an animal is fit for an intended journey and this may thereby impact animal welfare [10,11]. Lameness especially constitutes a significant animal welfare problem in regard to transportation, and it presents a challenge for the involved parties, when determining transport fitness in both cattle and pigs [2,10]. When assessing agreement between farmers, livestock drivers, and veterinarians on determining the transport fitness of lame cattle, agreement within and between the three groups was at best moderate [12]. This further shows the need for more specific guidelines to assist in the evaluation of the transport fitness of animals.

In the present study, all forensic case files on Danish pigs and cattle that had been declared unfit for transportation during a 10-year period (2014–2023) were evaluated with focus on the characterization of lesions that had rendered them unfit for transportation. In the study, the boundaries of the undefined conditions, i.e., “severe open wound” and “slightly injured or ill” in the EU Regulation [1], which must be addressed when evaluating transport fitness of pigs and cattle, are clarified.

## 2. Materials and Methods

In Denmark, all veterinary forensic cases are evaluated at the University of Copenhagen, and in the present study, case files concerning pigs and cattle that had been deemed unfit for transportation due to clinical manifestation, including indicators of stress and pain, by governmental veterinarians during the last 10 years (2014–2023) were examined. All cases had been reported to the police following transportation to slaughterhouses or transit locations all over Denmark. The case files, including photographs with rulers and casefile numbers, were evaluated and grouped according to the following specific conditions that had rendered them unfit for transportation: (1) the presence of rectal and/or vaginal prolapses, (2) the presence of umbilical and/or inguinal outpouchings (dimensions and +/− wounds) in pigs, (3) the presence of a “severe open wound”, (4) lameness rendering the animal unable to move independently without pain or to walk unassisted, and (5) animals being more than “slightly injured or ill”.

Based on the information in the case files, wounds were grouped according to size, age, and location in both pigs and cattle. Umbilical and inguinal outpouchings were grouped according to type and cause depending on whether the outpouching was wounded, had ruptured, or its size had rendered the pig unfit for transportation. Furthermore, the most common causes of lameness, the pathological manifestations found at necropsy and the age of these were characterized in both pigs and cattle.

## 3. Results

**Figure 1:**
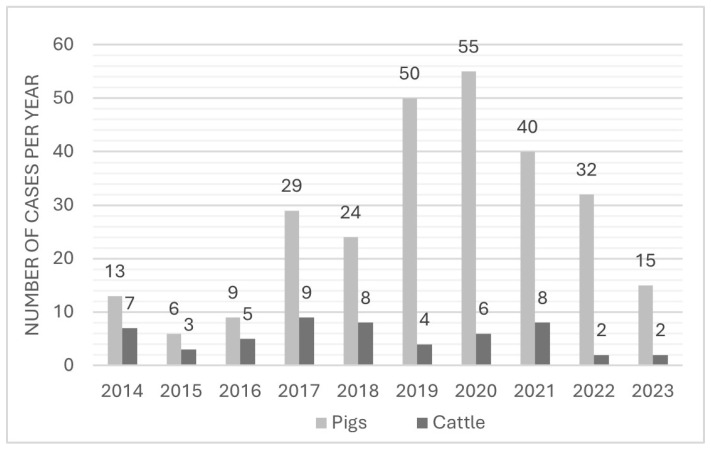
Number of forensic cases during a 10-year period (2014–2023) concerning pigs and cattle that had been deemed unfit for transportation.

A total of 327 case files, including 428 animals, of which 373 were pigs and 55 were cattle, had been submitted. Half of the cases (50%, 163/327) were from the 3-year period 2019–2021 (Figure 1) with the highest number of cases in 2020 (19%, 61/327). The cases had been reported from all parts of Denmark, with the most frequent police departments involved located in Jutland, e.g., North Jutland Police (38%, 124/327), Central and West Jutland Police (22%, 71/327), and South Jutland Police (18%, 58/327). The animals deemed to have been unfit for transportation were reported from 25 different Danish slaughterhouses and 4 transit locations.

### 3.1. Pigs

The majority of the cases concerned slaughter pigs (87%, 324/373), and they had predominately been transported in groups of 150–250 animals (63%, 236/373).

Wounds were the most frequent lesion rendering pigs (*n* = 249) unfit for transportation (Table 1), with a total of 253 wounds disclosed during the clinical examination.

**Figure 2:**
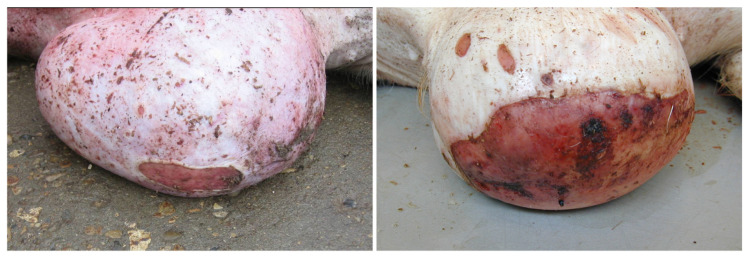
Umbilical outpouchings from two slaughter pigs with severe open wounds rendering the animals unfit for transportation.

**Figure 3:**
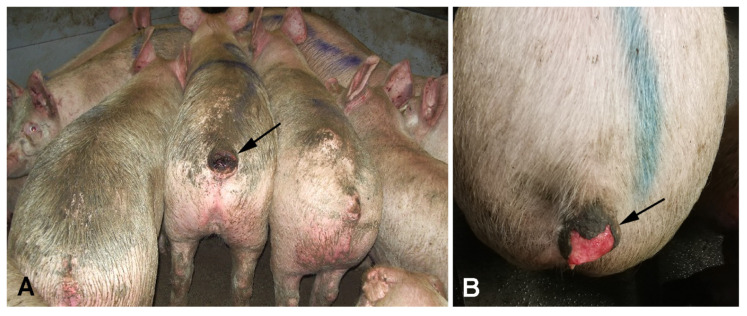
Two slaughter pigs (**A**,**B**) with severe open tail bite wounds (arrows), seen from behind (**A**) and seen from above (**B**), rendering them unfit for transportation.

**Figure 4:**
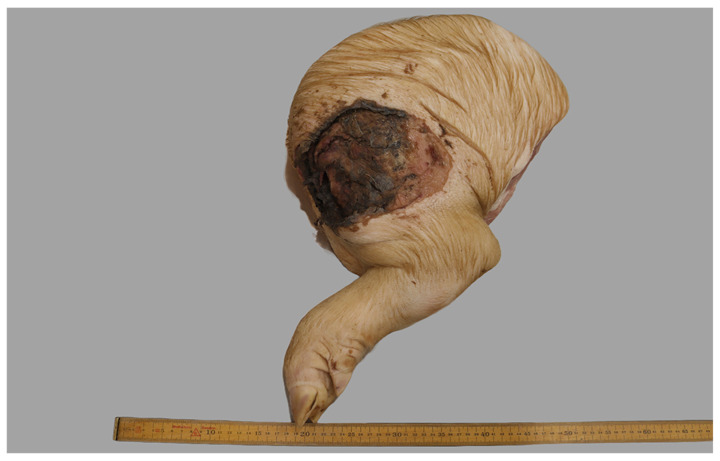
The left hindlimb of a slaughter pig, which had been transported to slaughter, was received for forensic evaluation. On the hindlimb a large, severe open wound was present. Ruler in cm.

The size of the wounds deeming pigs unfit for transport depended on the location. When located on umbilical and inguinal outpouchings (Figure 2) or tails (Figure 3), wounds < 3 cm in diameter (mean = 2.4 ± 0.6 cm) rendered the animals unfit for transport. By contrast, when present on other locations (e.g., shoulder region and on limbs; Figure 4) they had not been regarded as severe open wounds unless measuring >3 cm in diameter (Table 2). Moreover, the vast majority of the wounds (90%, 226/252), characterized at the pathological examination, were chronic.

**Table 2:** Location and size of wounds rendering pigs unfit for transportation.

Wound Location	Total Number of Wounds	Wound Size *	Number of Wounds
Tail	91	<3 cm in diameter	46 †
		3–5 cm in diameter	34
		>5 cm in diameter	11
Umbilical or inguinal outpouching	98	<3 cm in diameter	10
		3–5 cm in diameter	24
		>5 cm in diameter	60
Other locations ‡	63	<3 cm in diameter	0
		3–5 cm in diameter	15
		>5 cm in diameter	48

* In four of the ruptured hernias, it was impossible to determine the size of the wounds. † During the forensic examination, a tail wound was disclosed that had not been reported at the slaughterhouse. This wound was included. ‡ Includes shoulder region, hindquarters, limbs, ears, head, vulva, anus, flank, axillary region, and hoofs.

**Figure 5:**
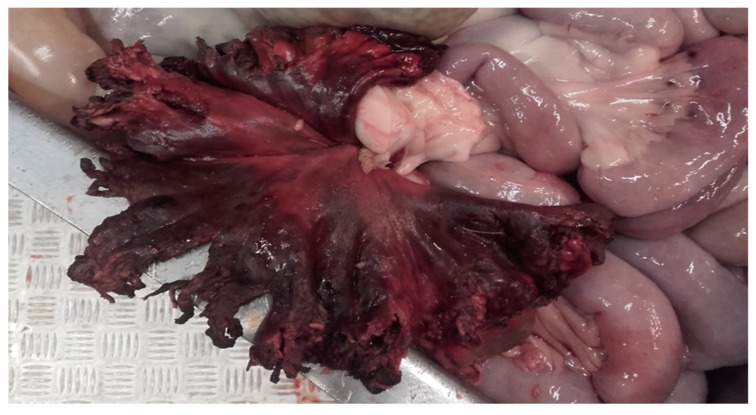
Material from a slaughter pig arriving at a slaughterhouse with a ruptured umbilical outpouching. The prolapsed abdominal material consisted of an acute hemorrhagic and necrotic segment of jejunum.

In 100 of the 106 pigs (94%) with an umbilical or inguinal outpouching, the underlying cause of the protrusions were the presence of a hernia or an enterocystoma. In the remaining cases, the outpouchings were due to an umbilical or inguinal abscess (4%, 4/106) or testicular infarction (2%, 2/106). Only 6 of the 106 pigs with outpouchings were reported due to the size of the outpouching (i.e., the large size causing complications such as restricted mobility), whereas the rest (100 pigs) had been deemed unfit for transportation due to the presence of a wound on the protrusion (Figure 2). Of the wounded outpouchings, six had ruptured with prolapse of abdominal material at arrival to the slaughterhouse (Figure 5). Furthermore, in two of the pigs reported at the slaughterhouse due to a wound on the outpouchings, a wound could not be identified at the forensic examination.

In animals stated clinically to be unfit for transportation as they were “unable to move independently without pain or to walk unassisted”, the pathological manifestations were primarily located in joints (55%, 69/125) affected by arthritis and arthroses (Table 3). However, a number of additional causes were also recorded; e.g., fractures and abscesses. Lesions causing lameness were in most cases chronic (i.e., with the presence of proliferative conditions including fibrosis and exostoses; 82%, 102/125).

**Figure 6:**
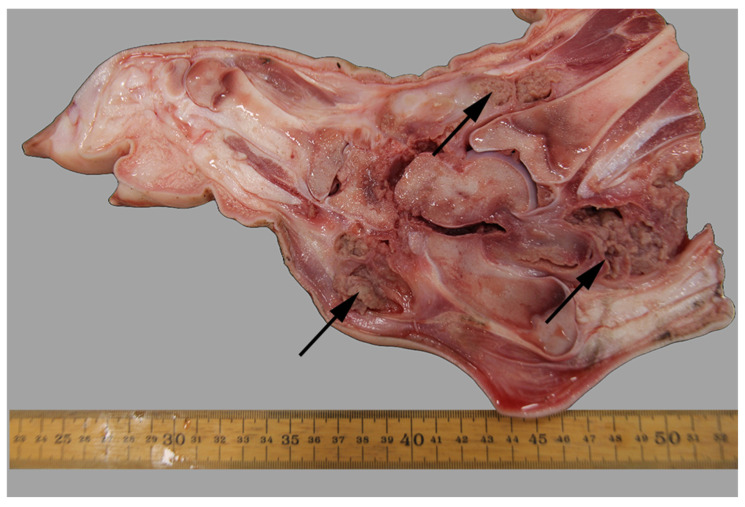
The right hindlimb of a slaughter pig deemed unfit for transportation due to lameness and a swollen hock joint was received for forensic examination. Following sagittal splitting, a diagnosis of chronic, proliferative, and ulcerative arthritis in the hock joint, with periarticular fibrosis and abscess formation (arrows), was established. Ruler in cm.

**Figure 7:**
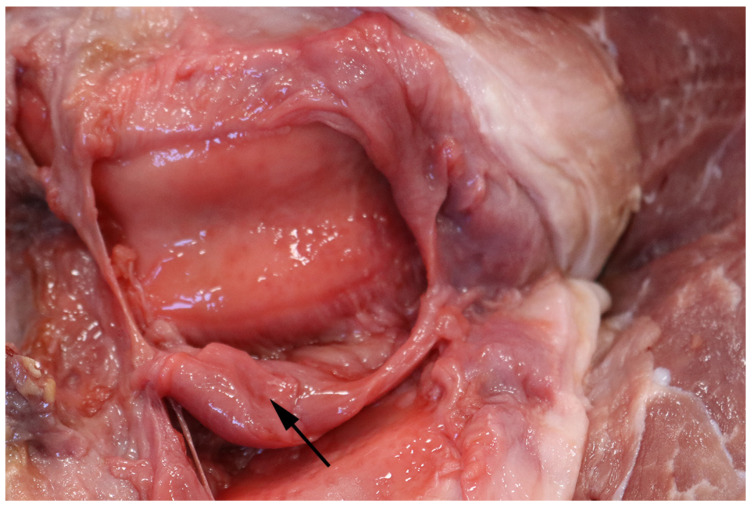
Opened knee joint from a slaughter pig with arthritis deemed unfit for transport. A pronounced proliferation of the synovial membrane was present (arrow).

**Figure 8:**
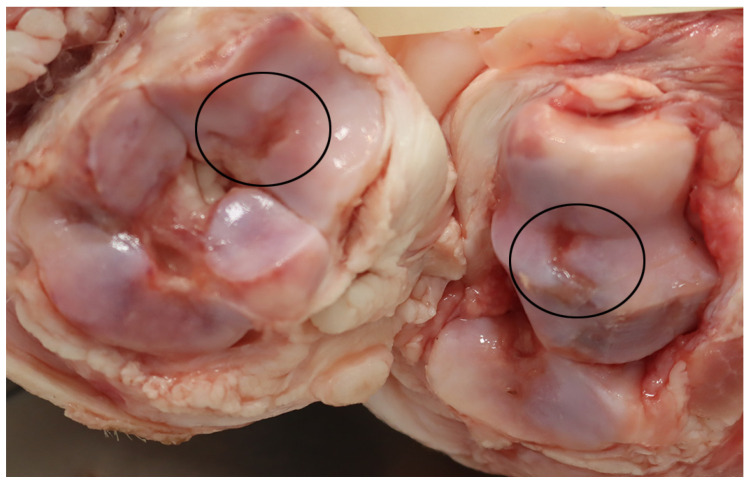
Opened hock joint from a slaughter pig deemed unfit for transport. At the forensic evaluation, a diagnosis of arthritis with proliferation of the synovial membrane, periarticular fibrosis and ulcerations in the joint cartilage (encircled) was established.

The joint lesions were most often present in the elbow or hock joints (63%, 46/73) (Figure 6). In pigs with arthritis the most common lesions consisted of proliferation of the synovial membrane (Figure 7) together with the synovium being mixed with an exudate (e.g., fibrin, serohemorrhagic fluid, pus, or a mixture of these). The area around affected joints was often swollen due to extensive periarticular granulation tissue formation, fibrosis (Figure 8), and in several cases osseous metaplasia, which, in the most severe cases, had caused ankylosis of the joints. In some cases, periarticular abscesses had developed with fistula rupture to the skin surface. Furthermore, erosions and ulcerations of the joint cartilage were often also present (Figure 8).

**Figure 9:**
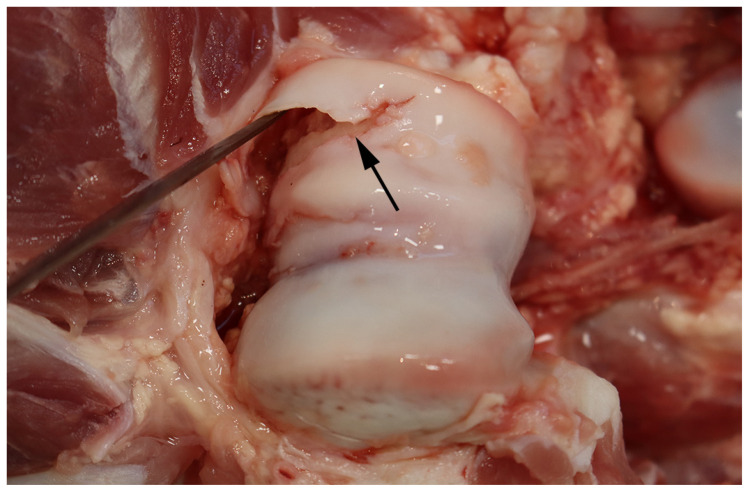
In the elbow joint of a lame slaughter pig deemed unfit for transportation, a dissecting lesion of the lateral humerus condyle was present (arrow).

In pigs with arthroses the primary lesions were erosions and/or ulcerations of the joint cartilage, often causing dissecting lesion deep into the cartilage (Figure 9). In most of these cases, the cause was osteochondrosis, and often multiple joints were affected in individual animals. Furthermore, a secondary proliferation of the synovial membrane and periarticular lesions in the form of fibrosis were also found in several of these cases.

Pathological manifestations rendering pigs unfit for transportation due to being more than “slightly injured or ill” included, e.g., elephantiasis (*n* = 6), auricular hematomas (*n* = 6), and chronic apostematous dermatitis of the mammae (*n* = 3).

### 3.2. Cattle

Almost half of the 55 cattle (45%) were between 2 and 5 years old, while the rest were divided almost equally at below 2 years of age (29%) and above 5 years of age (25%). The cattle had predominately been transported in groups of 10–30 animals.

The most frequent clinical condition rendering cattle unfit for transportation was lameness (64%, 35/55) (Table 4).

In animals stated clinically to have been “unable to move independently without pain or to walk unassisted”, the pathological manifestations were primarily fractures (48%, 21/44); however, several additional conditions had also rendered cattle unfit for transportation, e.g., joint lesions (arthritis; 20% and arthroses; 7%), hip luxation (7%), and abscesses (5%) (Table 5). The joint lesions were most often present in the knee, fetlock, and pedal joints (71%, 10/14). The lesions causing lameness were in far most cases chronic (93%, 41/44).

**Figure 10:**
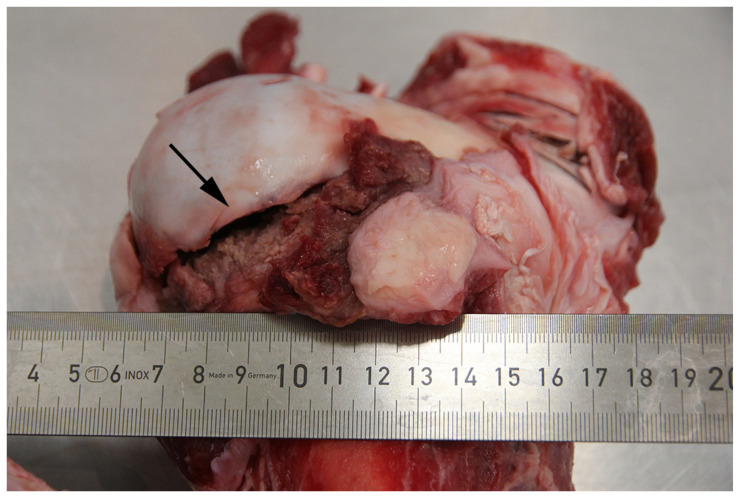
In a lame bull that had been transported for slaughter, a fracture (arrow) of the growth plate of the femoral head was disclosed at the forensic examination. Ruler in cm.

In the 21 animals with recorded fractures, more than half (57%, 12/21) were located on the femoral bone and often in the proximal epiphyseal area (i.e., causing epiphysiolysis caput femoris) (Figure 10). Of the remaining fractures 33% (7/21) were located on the ilium in the pelvis. The fractures were, in the vast majority of cases, closed, complete, and chronic, with callus formation and the presence of surrounding granulation tissue and fibrosis.

During the clinical examination of the 21 cattle with fractures, a common finding in 14 cases (67%) was the presence of an asymmetric pelvis.

**Figure 11:**
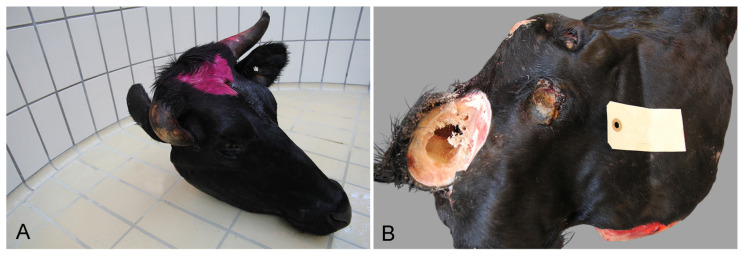
Head from a steer with an ingrown horn transported to slaughter (**A**). After removal of the horn, a severe open wound became visible on the right side of the head (**B**).

In 14 cases, cattle had been deemed unfit for transportation due to the presence of one or more wounds. At the forensic examination, another two heads of cattle were found to have a severe open wound linked to the clinical manifestations causing the animal to be considered unfit for transportation. Thereby, a total of 16 cases with severe open wounds were recorded. The wounds were present on multiple locations: hoofs (2/16), limbs (2/16), udder (2/16), throat (2/16), head (7/16), and tail (1/16). Except for three wounds caused by the ingrowth of a horn (Figure 11), the remaining wounds measured at least 3 cm in diameter, and 94% (15/16) of all wounds were chronic.

Pathological manifestations rendering cattle unfit for transportation due to being more than “slightly injured or ill” included, e.g., the ingrowth of a horn (*n* = 8), cachexia (*n* = 1), and ocular injury (*n* = 1).

## 4. Discussion

Farmers and livestock transporters have the responsibility to only transport animals fit for the intended journey, and they share the legal responsibility if a breach of the regulations occurs [1]. Therefore, it is problematic that even in cases where the legislation is very clear, unfit animals are still being transported. During the 10-year period (2014–2023), 16 pigs and 3 heads of cattle were transported with a rectal and/or vaginal prolapse even though these animals are clearly deemed unfit for transportation [1]. In a previous study, 94% of livestock drivers reported that they knew the rules regarding the fitness for transport of dairy cows well or very well, but only 52% could answer two questions correctly concerning relevant legislation. Furthermore, 72% had accepted to transport a cow even though they thought it was unfit. Also, conditions such as light, space, and time were reported to be occasionally insufficient for optimal evaluation of transport fitness by a number of respondents [11]. Therefore, the 19 animals observed with prolapses could have been overlooked, transported due to a lack of knowledge regarding the legislation, or accepted for transport by the transporter and/or farmer regardless of their physical/health condition. Also, the large group sizes of transported pigs, most being between 150 and 250 animals, is likely a contributing factor for animals with lesions being overlooked.

It is also surprising that in pigs most outpouchings (98, 92%) presented with a wound, which in Denmark clearly make them unfit for transportation. Six of the wounded outpouchings ruptured during transportation, showing the importance of this pathological condition in the evaluation of fitness for transportation. Moreover, pigs with wounds on umbilical outpouchings present a high prevalence (72%) of intra-abdominal lesions (e.g., hemorrhages, peritonitis, and adherences), emphasizing the fact that these animals are at risk of the worsening of their clinical condition when being transported [13]. However, it is tempting to speculate that pigs with outpouchings without wounds are also subjected to similar multiple intra-abdominal lesions.

In the EU Regulation the definition of a severe open wound is not clearly defined, nor are any guidelines offered on how to evaluate wounds [1]. When assessing a wound, the anatomical location, size, and stage of healing should be taken into consideration. Except for the wounds present on tails and umbilical or inguinal outpouchings in pigs, all wounds rendering both pigs and cattle unfit for transportation measured at least 3 cm in diameter. These thresholds are in accordance with the fact that disease progression, including wounds, reflects the impact on animal discomfort [14,15,16]. In future, this should be used as a point of reference for both farmers and livestock transporters when evaluating wounds on animals for an intended transport (i.e., in general, animals with wounds measuring 3 cm or more in diameter should not be transported). In pigs especially, the proximity to the underlying bones and joints in the tail makes it an “at-risk location” for wound complications (e.g., infections and osteomyelitis due to biting [17]). Therefore, smaller wounds at this location will in several situations render the animal unfit for transportation. Furthermore, smaller wounds (<3 cm in diameter) in combination with the ingrowth of a horn also rendered cattle unfit for transport. Therefore, all wounds should be evaluated carefully when deciding on transport fitness based on the location and cause of the wound.

Studies have shown that the presence of lameness is one of the most difficult clinical manifestations for farmers and livestock drivers to interpret with respect to transportation fitness [10,12,18]. Lameness was the condition that most often rendered cattle unfit for transportation in the present study, and fractures were found in 21 of the 35 lame cattle. The most common location of the fracture was in the proximal growth plate on the femur and in the pelvis. All the fractures were closed and could therefore not be seen when examining the animal, but an asymmetric pelvis had often been present. Therefore, attention should especially be paid to the hindlimbs as well as to the presence of a crooked pelvis when evaluating the transport fitness of lame cattle as this could indicate the presence of a fracture. Moreover, the worsening of the lameness that can occur during transportation must also be considered when examining a lame animal for an intended transport [19,20].

As mentioned, guidelines from private organizations [3,4,5,6,7] and public authorities [8] can be used when evaluating whether an animal should be considered (1) fit for transport, (2) fit for transport when certain precautions are taken, or (3) unfit for transport. However, and unfortunately, these guidelines differ in the examples provided to judge whether an animal is fit for transport in Denmark, when certain precautions are taken, or unfit for transport in Denmark. Arthritis in the hock joint is used as an example for both. Arthritis was also a prevalent cause of lameness in pigs, and lesions were most often present in the elbow or hock joints. This further emphasizes the need for more specific legislation on the transportation of animals.

## 5. Conclusions

In conclusion, with respect to the unfitness for transportation of pigs and cattle, animals with wounds measuring 3 cm or more in diameter should ideally not be transported; however, in “at-risk locations” even smaller wounds may render them unfit for transportation. Pathological manifestations rendering lame pigs and cattle unfit for transportation are different; in pigs the lesions are often located in the joints, whereas in cattle fractures dominated.

## Figures and Tables

**Table 1:** Clinical manifestations rendering pigs unfit for transportation.

Clinical Manifestations	Number of Pigs *
Wounds	249 †
Umbilical or inguinal outpouchings	106
Lameness	93
Prolapse	16
More than “slightly injured or ill”	27

* The same animal might have more clinical manifestations and more than one wound. † Two wounds reported at the slaughterhouse were not found during the forensic examination. These wounds are therefore not accounted for in Table 2.

**Table 3:** Pathological manifestations in pigs which had rendered them unfit for transportation due to lameness.

Pathological Manifestations	Number of Pigs †
Arthritis	41
Arthroses	28
Fractures	12
Limb hyperflexion/Tendon contracture	9
Other *	35

* Conditions such as hoof deformities, abscesses and subluxations. † The same animal might have more pathological manifestations.

**Table 4:** Clinical manifestations rendering cattle unfit for transportation.

Clinical Manifestation	Number of Cattle *
Lameness	35
Wounds	14
Prolapse	3
More than “slightly injured or ill”	11

* The same animal might have more clinical manifestations.

**Table 5:** Pathological manifestations in cattle that had rendered them unfit for transportation due to lameness.

Pathological Manifestations	Number of Cattle †
Fractures	21
Arthritis	9
Arthroses	3
Hip luxation	3
Other *	8

* Conditions such as abscesses, digital dermatitis, and overgrown hoofs. † The same animal might have more pathological manifestations.

## Data Availability

The data that support the findings of this study are available from the corresponding author upon reasonable request.
